# Are Si–C bonds formed in the environment and/or in technical microbiological systems?

**DOI:** 10.1007/s11356-023-28528-3

**Published:** 2023-07-24

**Authors:** Christoph Rücker, Magnus Winkelmann, Klaus Kümmerer

**Affiliations:** Institute for Sustainable Chemistry, Leuphana University Lüneburg, Universitätsallee 1, 21335 Lüneburg, Germany

**Keywords:** Dimethylsilanediol, Hexamethyldisiloxane, Decamethylcyclopentasiloxane, Octamethylcyclotetrasiloxane, Bond enthalpy, Biotrickling filter, GC retention time

## Abstract

Organosiloxanes are industrially produced worldwide in millions of tons per annum and are widely used by industry, professionals, and consumers. Some of these compounds are PBT (persistent, biaccumulative and toxic) or vPvB (very persistent and very bioaccumulative). If organosiloxanes react at all in the environment, Si–O bonds are hydrolyzed or Si–C bonds are oxidatively cleaved, to result finally in silica and carbon dioxide. In strong contrast and very unexpectedly, recently formation of new Si–CH_3_ bonds from siloxanes and methane by the action of microorganisms under mild ambient conditions was proposed (in landfills or digesters) and even reported (in a biotrickling filter, 30 °C). This is very surprising in view of the harsh conditions required in industrial Si–CH_3_ synthesis. Here, we scrutinized the pertinent papers, with the result that evidence put forward for Si–C bond formation from siloxanes and methane in technical microbiological systems is invalid, suggesting such reactions will not occur in the environment where they are even less favored by conditions. The claim of such reactions followed from erroneous calculations and misinterpretation of experimental results. We propose an alternative explanation of the experimental observations, i.e., the putative observation of such reactions was presumably due to confusion of two compounds, hexamethyldisiloxane and dimethylsilanediol, that elute at similar retention times from standard GC columns.

**Supplementary Information:**

The online version contains supplementary material available at 10.1007/s11356-023-28528-3.

## Introduction

**Scheme 1:**
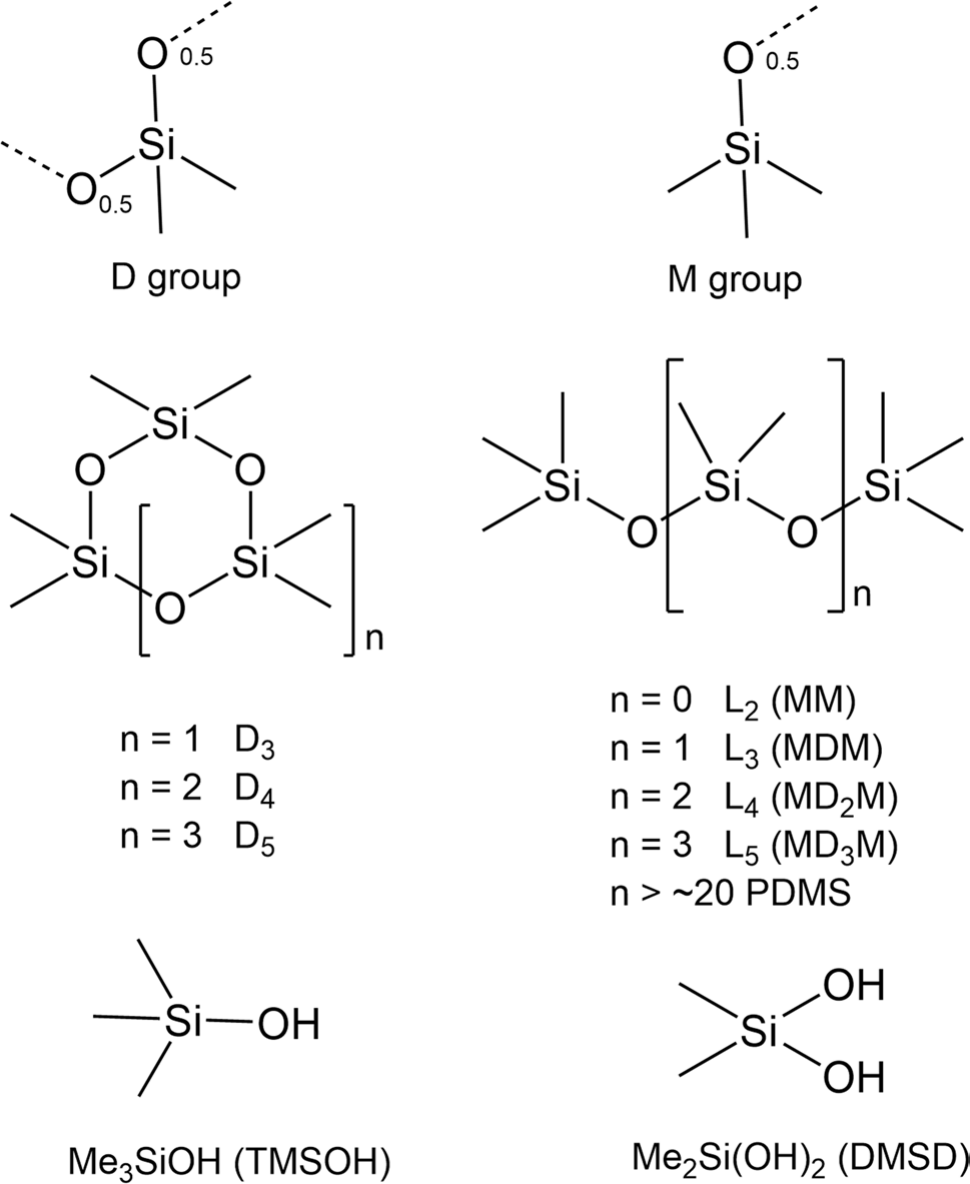
Molecular structures and shorthand designations of substructures and compounds mentioned in the text. D is a shortcut for the divalent Me_2_SiO_2/2_ substructure, M for the monovalent Me_3_SiO_1/2_ substructure

Organosiloxanes, compounds that contain C–Si–O subunits in their molecular structure, are man-made emerging ubiquitous pollutants now detected in many environmental compartments. Their structure is basically made of –Si–O– chains or rings, where each silicon atom additionally bears two organic moieties, in most cases methyl (CH_3_, Me) groups (–SiMe_2_–O–), occasionally higher or halogenated alkyl or phenyl groups. These compounds are produced worldwide in millions of tons per annum and are used by industry, professionals and consumers, so that they find their way into the environment (Rücker and Kümmerer 2015). In particular, octamethylcyclotetrasiloxane (D_4_), decamethylcyclopentasiloxane (D_5_), and dodecamethylcyclohexasiloxane (D_6_) are HPV (high production volume) chemicals according to the Organisation for Economic Co-operation and Development and the US Environmental Protection Agency, and are classified as PBT (persistent, bioaccumulative and toxic) and/or vPvB (very persistent and very bioaccumulative) under the European REACH regulation. These compounds are therefore substances of very high concern (SVHC) and are subjected to increasingly restrictive regulation, e.g., in Europe (ECHA 2018a, b, 2019, 2021; EU 2018, 2019). For structures and explanation of shorthand names of some important organosiloxanes, see Scheme 1.

For these reasons, degradation of organosiloxanes in the environment is an important issue. Complete degradation (mineralization) requires cleavage of both Si–O and Si–C bonds. Si–O–Si bond cleavage in organosiloxanes by hydrolysis to produce silanols is slow in most cases but is catalyzed by acid, base, or Lewis acid (Xu 1998; Xu et al. 1998, 2017; Ducom et al. 2013; Gatidou et al. 2016; ECHA database www.echa.europa.eu). In contrast, the only well-documented environmental Si–CH_3_ cleavage is by reaction with OH radicals in the atmosphere (Alton and Browne 2020, 2022), whereby in a series of reaction steps not known in detail a Si–CH_3_ is replaced by a Si–OH substructure. Repetition of this sequence in combination with hydrolysis will finally lead to silicic acid, Si(OH)_4_, or its oligomers or its polymeric anhydride silica, SiO_2_. Reaction with OH radicals may occur also in water (Xu et al. 2017; Han et al. 2020).

Since Si–C bonds do not occur in living nature, it is still an open question whether there exist microorganisms able to cleave such bonds (Rücker and Kümmerer 2015; Petkowski et al. 2020; Rücker et al. 2023).

During the last 20 years, degradation of organosiloxanes has become a hot research topic in the context of biogas valorization (Braganca et al. 2020; Golmakani et al. 2022; Rivera-Montenegro et al. 2022; Rücker et al. 2023). Biogases, mixtures of mostly methane and carbon dioxide, are an upcoming power resource advantageous in terms of greenness, sustainability, and climate protection. There are three kinds of biogases (Rasi et al. 2011), those from anaerobic digestion of wastewater treatment sludge (Bougrier et al. 2006; Appels et al. 2008; De Arespacochaga et al. 2020), those released from landfills (Bolan et al. 2013; Kuhn et al. 2017; Li et al. 2019; De Arespacochaga et al. 2020; Braganca et al. 2020; Wang et al. 2020), and those produced from agricultural wastes (Rasi et al. 2013; Akhavan et al. 2014; Foppiano et al. 2020; Oliveira et al. 2022). Digester gas contains siloxanes that were adsorbed to sludge out of sewage, coming from use of personal care products containing, e.g., D_4_ and D_5_. Landfill gas contains siloxanes that probably are hydrolysis products of polydimethylsiloxanes (PDMS) present, e.g., in waste building materials, or residues in not quite empty containers of personal care products. Even in some biogases from agricultural waste traces of siloxanes of unknown origin were detected. Whenever biogas is combusted, e.g., for power generation, siloxanes therein are transformed into solid SiO_2_ particles that cause serious damage of equipment (Gersen et al. 2019; De Arespacochaga et al. 2020) and may threaten human health. Fuel cells are extremely sensitive to siloxane traces in feed biogas (Madi et al. 2015; Papurello and Lanzini 2018). Therefore, siloxanes have to be removed from crude biogas, a task of immediate economic relevance (Golmakani et al. 2022). For this purpose, adsorption to porous materials such as active carbon is state of the art, but alternative technologies are in high demand (De Arespacochaga et al. 2020; Braganca et al. 2020; Gaj 2020; Das et al. 2022; Rivera-Montenegro et al. 2022; Alves et al. 2023; Pascual et al. 2023; Gaj and Cichuta 2023). As such, recently biodegradation of siloxanes is an active field of research (Grabitz et al. 2021; Rücker et al. 2023). For example, in a biotrickling filter (BTF) a gas stream is brought into intimate contact with biodegrading microorganisms in the presence of an aqueous mineral solution. For exploring this technology, N_2_ or air were spiked with siloxane traces (D_4_ and/or D_5_) to serve as model gases in laboratory-size BTFs (Santos-Clotas et al. 2019, 2020; Boada et al. 2020; Pascual et al. 2020, 2021a).

Despite all uncertainty on details of degradation, for many years, it was generally accepted that if Si–CH_3_ bonds in siloxanes are at all reactive in the environment, then methylsiloxanes will finally be oxidatively degraded into silica, carbon dioxide and water. This view was and is plausible in our environment rich in oxygen and is based on thermodynamics that favor Si–O over Si–C moieties due to the Si–O bond being far stronger than the Si–C bond. This is of course in line with the usual environmental mineralization of organic pollutants into carbon dioxide and water.

**Scheme 2:**
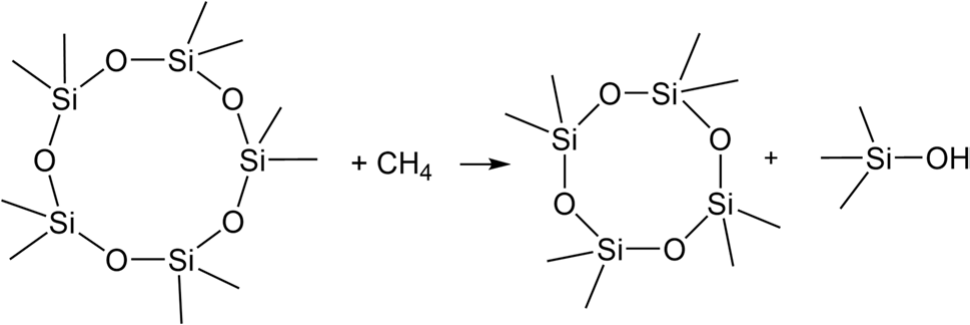
Detailed view of putative reaction (1)

All organosiloxanes are industrially synthesized in a sequence comprising as the first step the Müller–Rochow process, in which Si–CH_3_ bonds are formed from elemental silicon and chloromethane (2 CH_3_Cl + Si → (CH_3_)_2_SiCl_2_), requiring harsh conditions (300 °C, 3 bar) even under CuO catalysis (Beck et al. 2022). It was therefore very unexpected and contra-intuitive when in 2014 Tansel and Surita declared formation of new Si–CH_3_ bonds from siloxanes and methane thermodynamically favorable, and moreover suggested such reactions to occur under the mild conditions in landfills or digesters, for example hypothetical reaction (1) (Scheme 2) (Tansel and Surita 2014).

1 $$\begin{array}{c}{\mathrm{D}}_{5}+\mathrm{methane}\to {\mathrm{D}}_{4}+{\mathrm{Me}}_{3}\mathrm{SiOH}\\{\mathrm{C}}_{10}{\mathrm{H}}_{30}{\mathrm{Si}}_{5}{\mathrm{O}}_{5}+{\mathrm{CH}}_{4}\to {\mathrm{C}}_{8}{\mathrm{H}}_{24}{\mathrm{Si}}_{4}{\mathrm{O}}_{4}+{\left({\mathrm{CH}}_{3}\right)}_{3}\mathrm{SiOH}\end{array}$$

This hypothetical reaction comprises formation of a Si–CH_3_ bond under cleavage of a Si–O bond. Tansel and Surita estimated the free energy of this reaction as − 251 kJ/mol, meaning an *exothermic*, thermodynamically favored reaction. This suggestion did not find much resonance until 2020, when Zhang et al. reported on an experiment in which for the first time they treated a real biogas containing traces of D_5_ and D_4_ in a (micro)aerobic BTF (Zhang et al. 2020). Most surprisingly to us, they interpreted their experimental results in terms of reaction (1), reporting formation of L_2_, a condensation product of Me_3_SiOH, as the major product. Such an accidental Si–CH_3_ bond formation seems questionable in view of what is known of pertinent bond enthalpies, see Table 1 that represents the current knowledge of organosilicon chemistry, obtained from a large body of experimental evidence. Zhang et al.’s interpretation contradicts general experience of organosiloxane behavior in the environment (Rücker and Kümmerer 2015; Homem and Ratola 2020; Rücker et al. 2023), and would have far-reaching consequences not only for biogas purification, but also for understanding the fundamental chemistry of organosilicon compounds, and last not least for industrial organosiloxane synthesis. Therefore, the putative Si–CH_3_ bond formation under mild conditions deserved a detailed investigation, the more so since it seems to be gaining acceptance as shown by the following quotations: “… the likely formation of L2 as an intermediate metabolite of D4 degradation” (Pascual et al. 2021b, no reference given), and “methane utilization may be a possible reaction mechanism for trimethylsilanol formation” (Xiang et al. 2021, referencing Tansel and Surita 2014). These authors obviously took for granted the possibility of Si–CH_3_ bond formation under mild conditions. The title question is asked here for the first time, to the best of our knowledge. The objectives of the present work were (i) to decide whether Si–C bond formation from siloxanes and methane under mild condition is real or erroneous, (ii) in the latter case, to understand what really may have happened in Zhang et al.’s experiment. So, we investigated the issue by scrutinizing the Tansel and Surita (2014) paper (“Consideration of reaction enthalpy” section) and the Zhang et al. (2020) paper (“Consideration of experimental evidence” section). From that a more realistic explanation of Zhang et al.’s experimental results evolved as a hypothesis that was shown to be in concordance with current knowledge (“Test of hypothesis” section).

**Table 1:** Various sets of average bond enthalpies (kJ/mol)

	Set 1^a^	Set 2^b^	Set 3^c^	Set 4^d^	Set 5^e^	Set 6^f^
Si–O	368	444	368	477–549	452	557
Si–C	301	306	290	369–376	318	385
C–H	413	416	416	420	411	412
O–H	463	463	463^ g^	463^ g^	463^ g^	498

^a^Tansel and Surita (2014), referencing Wilbraham et al. (2008)

^b^Riedel and Janiak (2022)

^c^Clayden et al. (2012)

^d^Brook (2000)

^e^Sarai et al. (2021)

^f^Zhu and Zhou (2022)

^g^Taken from sets 1 and 2, since not available in sets 3–5

## Consideration of reaction enthalpy

Tansel and Surita (2014) roughly estimated reaction enthalpies as differences between enthalpies of products and reactants. Neglecting any ring strain effects or entropy terms, the enthalpy of a compound was expressed as the sum of its (average) bond enthalpies. This is equivalent to a reaction enthalpy being the difference of bond enthalpies on the left- and on the right-hand side of a reaction equation, or the difference of enthalpies of bonds formed and bonds broken. Attempting to reproduce their calculations, we followed the same approach, counting the bonds on the left-hand side of Eq. (1), 10 Si–O, 10 Si–C, 34 C–H bonds, and on the right-hand side, 9 Si–O, 11 Si–C, 33 C–H, 1 O–H bonds (Scheme 2). As a result, in this reaction one Si–O and one C–H bond are broken and one Si–C and one O–H bond are newly formed.

There are several sets of average bond enthalpies available; six sets are shown in Table 1. Using the set of bond enthalpies given in Tansel and Surita’s paper (set 1), we find reaction (1) to be *endothermic* by 17 kJ/mol, in obvious contradiction to their result of 251 kJ/mol exothermicity. If alternatively set 2, 3, 4, or 5 is used, reaction (1) turns out to be endothermic by 91, 31, 58–137, or 82 kJ/mol, respectively. Set 6 is the result of recent high-level ab initio calculations (Zhu and Zhou 2022); using these numbers, reaction (1) is endothermic by 86 kJ/mol. According to all these six sets, this hypothetical reaction is more or less endothermic and therefore not very likely to occur spontaneously or under catalysis to a reasonable extent. This is understandable since a very strong Si–O bond is broken in favor of a less strong Si–C bond, while the O–H bond formed, though stronger than the broken C–H bond, is unable to overcompensate.

If we additionally take into account ring strain of cyclosiloxanes, the conclusion is even stronger since ring-shrinking will build up further strain. Unfortunately, the only available numerical values of ring strain in D_*n*_ are rather old and contradictory. The most recently published values of strain energies of D_3_, D_4_ and D_5_ are 10.5, 1.01, and 1.05 kJ/mol, respectively (Voronkov 1996). Thereby an effect on reaction (1) is negligible, while the analogous hypothetical reaction (2) below, where D_3_ is formed from D_4_, suffers from an additional ring strain effect of 9.5 kJ/mol. Using older ring strain estimates for D_3_, D_4_, and D_5_ (80, 60, and 40 kJ/mol, Voronkov et al. 1991), reactions (1) and (2) are both disfavored by an additional ring strain effect of 20 kJ/mol.

Similar discrepancies are found between Tansel and Surita’s vs our calculations for all other hypothetical reactions mentioned in their paper. One obvious error in their calculations is the number of Si–C bonds in Me_3_SiOH given in their Table 2 as 1 instead of 3. Due to this error, all their calculations for reactions involving Me_3_SiOH (including reaction (1)) must be considered erroneous. However, even after correction of this error we are still unable to reproduce the G values given in their Table 2, we suspect that these numbers resulted from actually using bond energies other than those in set 1. For example, in their Fig. 3a and c, the Si–O bond energy is depicted about 450 kJ/mol, strangely with the same source given as that for set 1. Finally, in calculating the ΔG values for reactions (their Table 3, based on the numbers in their Table 2), the plus/minus signs or the directions of reactions seem to be confused.

## Consideration of experimental evidence

In Zhang et al.’s (2020) experiment, a stream of biogas from an anaerobic digester (mostly CH_4_ and CO_2_) containing traces of D_5_, D_4_, and H_2_S was mixed with air (final O_2_ concentration 1%) and was treated at 30 °C in a BTF (Zhang et al. 2020). The BTF contained a circulating aqueous mineral nutrient solution and a biofilm (developing from an anaerobic digested sludge inoculum) and was continuously run for six months. Thereby, the H_2_S in the biogas was oxidized to sulfuric acid, the pH fell to 1.5, removal of H_2_S from the gas stream was almost complete (> 95%) while removal of siloxanes was partial (D_5_ removal up to 52%). At day 180, the aqueous reaction broth was analyzed for organosiloxanes by extraction into THF followed by GC/MS analysis using a 60 m HP-5MS capillary GC column. The gas chromatogram (shown in Zhang et al. (2020) as Fig. 7, red trace) showed a major product (retention time ~ 11.28 min) and a minor product (retention time 12.89 min) that the authors interpreted as L_2_ (hexamethyldisiloxane) and D_3_ (hexamethylcyclotrisiloxane), respectively. However, both these attributions are problematic.

Interpretation of the major product as L_2_ is subject to doubt for at least two reasons.

**Scheme 3:**
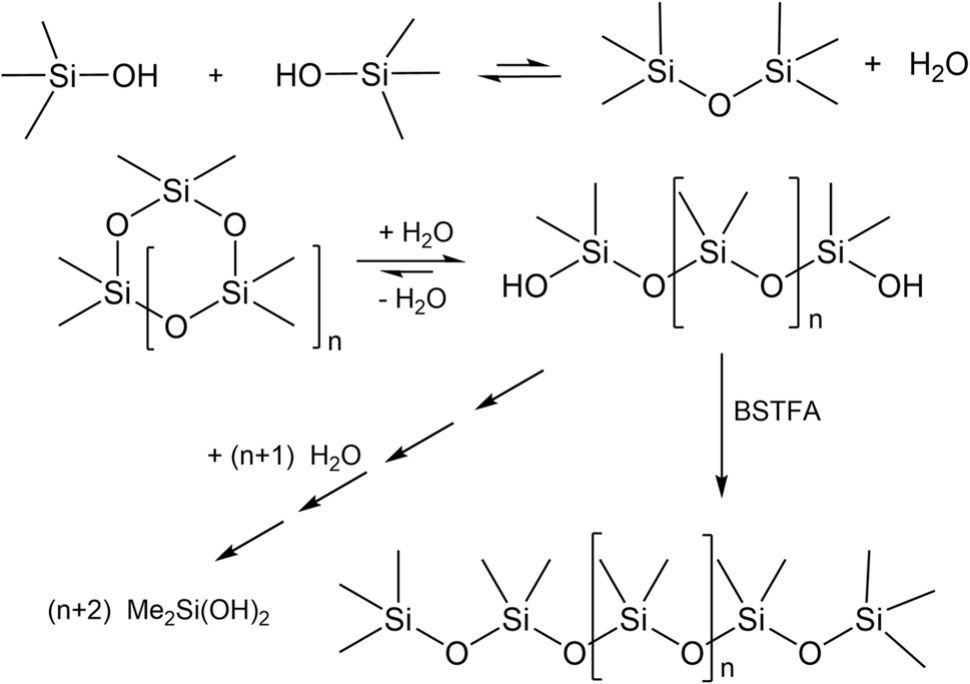
Hydrolysis equilibria of L_2_ and cyclic methylsiloxanes, and silylation of oligomeric siloxanediols (oligomerdiols)

First, it is known that L_2_ suffers hydrolysis according to the equation Me_3_SiOSiMe_3_ + H_2_O $\rightleftharpoons$ Me_3_SiOH + HOSiMe_3_ (Scheme 3). This is a hydrolysis equilibrium dominated in dilute aqueous solution by Me_3_SiOH (Spivack and Dorn 1994), and the experimental half-life of L_2_ in aqueous solution due to this reaction is ~ 4 days at pH 7 and 25 °C, and ~ 1.5 h at pH 5 and 25 °C (ECHA database www.echa.europa.eu). This fact alone renders presence of L_2_ in any reasonable amount at the end of Zhang et al.’s experiment (aqueous solution, pH down to 1.5, 30 °C, 180 days) extremely unlikely.

Second, the starting siloxanes D_4_ and D_5_ as well as their hydrolysis products and product D_3_ are made of D groups exclusively, whereas L_2_ (MM) consists of M groups.

To explain the putative formation of L_2_ (via condensation of Me_3_SiOH, the reverse of the above hydrolysis), Zhang et al. postulated the occurrence of reactions, called by them “ring-shrinking polyreactions”, that had been theoretically proposed by Tansel and Surita (2014), their “methane consuming siloxane reactions”:

1 $${\mathrm{D}}_{5}+{\mathrm{CH}}_{4}\to {\mathrm{D}}_{4}+{\mathrm{Me}}_{3}\mathrm{SiOH}$$

2 $${\mathrm{D}}_{4}+{\mathrm{CH}}_{4}\to {\mathrm{D}}_{3}+{\mathrm{Me}}_{3}\mathrm{SiOH}$$

3 $${\mathrm{D}}_{3}+{3\,\mathrm{CH}}_{4}\to 3\;{\mathrm{Me}}_{3}\mathrm{SiOH}$$

The essence of reactions (1)–(3) is formation of a third Si–C bond at a silicon atom that is already engaged in two Si–C bonds, under cleavage of a Si–O bond, that is, formation of M from D groups and methane. These reactions according to Tansel and Surita are exothermic, ΔG^0^ =  − 251 kJ/mol for both (1) and (2), and − 753 kJ/mol for (3), see, however, the “Consideration of reaction enthalpy” section above.

Additionally, Zhang et al. treated the crude product mixture (THF extract after drying with anhydrous Na_2_SO_4_) with bis(trimethylsilyl)trifluoroacetamide (BSTFA) in order to transform silanols into their trimethylsilyl derivatives for better GC/MS behavior (Scheme 3). The gas chromatogram after that treatment (shown in Zhang et al. (2020) as Fig. 7, blue trace) showed a peak of medium intensity at retention time 11.28 min, a peak of high intensity at 15.12 min, and peaks at 21.86 (low intensity) and 28.08 min (minute intensity). Zhang et al. (2020) attributed these peaks to L_2_, L_3_, L_4_ and L_5_, respectively. The latter three compounds presumably were the silylation products of dimethylsilanediol (DMSD), tetramethyldisiloxane-1,3-diol (dimerdiol), and hexamethyltrisiloxane-1,5-diol (trimerdiol), all these silanols are hydrolysis products of D_4_ and D_5_ (Scheme 3). There is another serious problem in this data interpretation, the absence of DMSD in the crude reaction mixture before silylation, where according to the silylation result it should be the dominant product.

In view of these problems, Zhang et al.’s data interpretation seemed highly questionable. Our hypothesis is that the major peak in the GC trace of Zhang et al.’s crude THF extract (retention time close to 11.28 min) does not correspond to L_2_ but to Me_2_Si(OH)_2_ (dimethylsilanediol, DMSD), whereas the peak at 11.28 min in the silylated sample does correspond to L_2_. In other words, L_2_ and DMSD may appear at similar retention times on Zhang et al.’s GC column and therefore may have been confused.

Our hypothesis is based on facts:

- D_4_ and D_5_ in acidic aqueous solution are readily hydrolyzed to mostly DMSD, with trimerdiol and dimerdiol being obvious intermediates (Spivack and Dorn 1994; Kozerski and Durham 2006; ECHA database www.echa.europa.eu).
- DMSD is extracted into THF from aqueous solution (Varaprath et al. 1998, 2006).
- DMSD is analyzable by GC without decomposition, Table S1 in the Supplementary Material presents retention times of DMSD measured on various capillary GC columns under various conditions.

If our hypothesis is correct, the problems mentioned above are resolved, and questionable reactions such as (1)–(3) are not required to explain Zhang et al.’s experimental results.

For the presence of L_2_ in the silylated sample, there are several possible explanations. (i) Silylation by BSTFA of a water trace still present in the dried THF extract or newly diffused into it during handling in laboratory air, due to THF’s hygroscopicity. (ii) L_2_ contamination in reagent BSTFA due to moisture contact during its production, storage or handling. (iii) Traces of Me_3_SiOH may be present in the real biogas in concentration too low to be detected, are enriched in the reaction broth by continuous entry during the 180 day experiment, are extracted into THF and then silylated by BSTFA resulting in L_2_.

## Test of hypothesis

Our hypothesis essentially is the possibility of confusing L_2_ and DMSD if GC retention times only are considered. To test this hypothesis, the GC retention times of both compounds should be known under identical conditions. The type of GC column used for such a comparison should be similar (not necessarily the same) to that used by Zhang et al. (2020).

Table S1 in the Supplementary Material presents all retention times of DMSD that we were able to find in the literature reported up to mid-2022. Table S2 is a similar compilation for L_2_. These measurements were performed using various column materials, various column dimensions and under various conditions. A single paper provides GC retention times for both DMSD and L_2_ (3.02 and 3.88 min, respectively), but capillary columns used and conditions were not specified and may have been different for the two compounds (Varaprath 1999). Measurements using the most prevalent column material (5% diphenyl 95% dimethylpolysiloxane, the one used by Zhang et al.) and the most common column length (30 m) are highlighted in Tables S1 and S2 by italics. Restricting attention to these reports, retention times of DMSD still extend from 2.43 to 4.3 min, those of L_2_ vary between 1.65 and 6.4 min depending on conditions (temperature, carrier gas flow, solvent etc.) in a not quite transparent manner. Though the retention time range for DMSD is completely covered by that for L_2_, this data alone does not mean that under a particular set of conditions the retention times of both compounds are similar. However, in a most recent article (Geng et al. 2022) retention times of DMSD and L_2_ under identical conditions were published for the first time, 7.86 min for DMSD and 7.65 min for L_2_ (TG-5 column, 60 m × 0.25 mm × 0.25 μm, 1.00 mL/min He, temperature program 3 min at 40 °C, 5 °C/min up to 90 °C). This result (2.7% difference in retention times) strongly supports our hypothesis. Similarly, preliminary measurements of retention times of both compounds in our lab resulted in 4.54 min for DMSD and 3.88 min for L_2_ (Optima 5MS Accent capillary column (30 m × 0.25 mm × 0.25 μm), 1.5 mL/min He, temperature program 5 min at 35 °C, 10 °C/min up to 70 °C, 30 °C/min up to 260 °C) when running a mixture of both compounds dissolved in THF (M. Winkelmann, unpublished). This result (17% difference in retention times) is at least compatible with our hypothesis.

## Discussion

Meanwhile, Zhang et al. in a control experiment monitored the transformation of D_5_ in dilute sulfuric acid (pH 2.0) at 30 °C for 30 days, conditions similar to those of their original experiment but without any added inoculum. The major observed product GC peak eluted at a retention time close to that of the major product in the original experiment, Zhang et al. now interpreted it as DMSD, though they did not report a mass spectrum of this peak. Consequently, they withdrew their claim for L_2_ formation, and thus for Me_3_SiOH and Si–C bond formation in their BTF (Zhang et al. 2021a). They also revoked a one-step reaction D_5_ → MD_3_M (L_5_) that appeared in the Graphical Abstract of their 2020 paper without any comment and that again comprised formation of M from D groups. In another subsequent study they confirmed that D_5_ removal in such an acidic BTF is due to acid-catalyzed hydrolysis (Zhang et al. 2021b). However, Zhang et al. (2021a, b) did not discuss reasons for the original erroneous claim.

As an aside, Zhang et al.’s identification of their original minor product (peak at 12.89 min) as D_3_ is also doubtful. D_3_ is subject to rapid hydrolysis as well (t_1/2_ = 2 min at pH 4, 23 min at pH 7, 25 °C, ECHA database www.echa.europa.eu; Gerhards et al. 2022). So the minor product probably was hexamethyltrisiloxane-1,5-diol (trimerdiol), which in the subsequent silylation step gave rise to L_5_. Mass spectra of D_*n*_ and the corresponding *n*-merdiol under EI (70 eV) ionization are nearly identical (Varaprath and Lehmann 1997; Varaprath et al. 2006).

An anonymous reviewer requested us to “comment on the hydrophilicity of each bond”.

Hydrophilicity of a compound roughly parallels the polarity of a molecule that in turn is the vector sum of polarities of all bonds present. In a simple siloxane, the only highly polar bonds are the Si–O bonds. Numerically, with the electronegativities of C, Si, O, and H being 2.55, 1.90, 3.44, and 2.20, respectively, the bond polarities (electronegativity differences of atoms) are as follows: Si–O 1.54, Si–C 0.65, C–H 0.35 (dimensionless). In a cyclic siloxane the bond dipole vectors add to zero, and in a linear siloxane nearly so. So, siloxanes (unless carrying highly polar functional groups) are nonpolar and therefore hydrophobic compounds, as described by the octanol/water partition coefficients K_OW_ or log K_OW_. The experimental log K_OW_ values of D_3_ (4.4), D_4_ (6.89), D_5_ (8.07), and Me_3_SiOH (1.19) (ECHA database www.echa.europa.eu) mean that the cyclic siloxanes are extremely hydrophobic, and even Me_3_SiOH prefers the octanol phase to the water phase by a factor of 15. Thus, hypothetical reaction (1) would transform extremely hydrophobic compounds into somewhat less hydrophobic ones.

An anonymous reviewer suggested a discussion of the influence of “super/sub-critical waters and also the energy provided by explosion of micro/nano-bubbles formed during some reactions”.

Hydrolysis of siloxanes (that is, Si–O bond cleavage) strongly depends on temperature, for example, hydrolyses of D_4_ and D_5_ are 22 and 17 times faster at 35 °C than at 10 °C (ECHA database www.echa.europa.eu). In hydrolysis experiments of D_*n*_ or L_*n*_ in water at these moderate temperatures Si–C cleavage or Si–C formation was never observed. However, in supercritical water (390 °C, 27 MPa), several organosilicon compounds including siloxanes underwent Si–C cleavage (Itami et al. 2004). Silicone wastes under subcritical or supercritical water conditions (250–390 °C, 50–280 bar) were even reported to decompose down to CH_4_ and/or a solid inorganic material containing SiO_2_ (Ulbrich et al. 2022).

Low-density polyethylene crosslinked with siloxane moieties, on the other hand, when treated with supercritical water (~ 350 °C), lost the crosslinks by cleavage of siloxane Si–O bonds; additionally, polyethylene chains were cut (Goto et al. 2008; Baek et al. 2016; Elmanovich et al. 2022). In all these experiments, no hints to formation of Si–C bonds were reported. Moreover, supercritical or very hot subcritical water does not play a role in landfills, digesters, biotrickling filters or in the free environment, to the best of our knowledge.

Micro/nanobubble technology is an emerging opportunity in wastewater treatment (Nair et al. 2022; Zhou et al. 2022a, b); however, no information on the behavior of siloxanes under such conditions seems to be available.

## Conclusion and outlook

Scrutiny of the pertinent papers clearly shows that there is no evidence for formation of Si–C bonds from methane and siloxanes under mild conditions, neither theoretical nor experimental. The calculations put forward in favor of such reactions are incomprehensible, while the only case of such reactions putatively observed (in a biotrickling filter) most likely was erroneous, in that a product GC peak corresponding to DMSD seems to be misinterpreted as corresponding to L_2_. On a slightly polar GC column L_2_ and DMSD elute at similar retention times, so that they may easily be confused if retention times only are considered. This explains Zhang et al.’s (2020) experimental observations, and thus both objectives of our work were reached.

Reactions such as (1)–(3) should be favored by conditions in the technical microbiological systems discussed here (landfills, anaerobic digesters, biotrickling filters for biogas cleaning): Siloxane substrates and methane and bacteria are present in relatively high concentrations for durations long enough to allow for adaptation and selection of microorganisms. If under these conditions such Si–C bond forming reactions do not occur, then their occurrence under the less favorable conditions in the environment (in the field) is even less probable.

According to a recent review (Braganca et al. 2020), in analyses of biogases the presence of L_2_ was often reported, that of Me_3_SiOH sometimes, while Me_2_Si(OH)_2_ (DMSD) was not only never reported, but also never looked for. This is surprising since at least in cases of proven Me_3_SiOH formation by hydrolysis of oligo- or polymeric linear dimethylsiloxanes (PDMS), formation of large amounts of Me_2_Si(OH)_2_ should be expected. Moreover, the major hydrolysis product of cyclic dimethylsiloxanes such as D_4_ or D_5_ also is Me_2_Si(OH)_2_, a compound both well-soluble in water and volatile. As expected, in leachates from waste deposit sites both Me_3_SiOH and Me_2_Si(OH)_2_ were found (Grümping and Hirner 1999). So, it seems possible that L_2_ reported in biogases at least in some cases or in part actually was DMSD.

## Supplementary Information

Below is the link to the electronic supplementary material.

Supplementary file1 (PDF 658 KB)

## Data Availability

All data and materials are included in the main text and the Supplementary Material.
