# Supplementary material for: Are Si–C bonds formed in the environment and/or in technical microbiological systems?
Source: Environ Sci Pollut Res Int. 2023 Jul 24;30(39):91492–500. doi: 10.1007/s11356-023-28528-3 (PMC10439844; doi:10.1007/s11356-023-28528-3)
Supplement: Supplementary file 1 — Supplementary file1 (PDF 658 KB) [file 11356_2023_28528_MOESM1_ESM.pdf]

Supplementary Material to

Are Si–C bonds formed in the environment and/or in technical microbiological systems?

Christoph Rücker*, Magnus Winkelmann, Klaus Kümmerer

Institute for Sustainable Chemistry, Leuphana University Lüneburg,

Universitätsallee 1, D-21335 Lüneburg, Germany

e-mail: christoph.ruecker@leuphana.de

Table S1. GC columns, conditions and retention times of DMSD

**GC Column material, dimensions** (m x mm x µm)	**Carrier gas, flow rate** (mL/min)	**Temperature program**	**Solvent**	**DMSD retention time (min)**	**Reference**
DB-1,<br>60 x 0.246 x 0.25	He,<br>n.a.	70 °C 3 min,<br>15 °C/min up to 250 °C	THF	4.9	Lehmann et al. (1994)
HP-5 MS,<br>*30 x 0.25 x 0.25*	He,<br>n.a.	70 °C 3 min,<br>20 °C/min up to 310 °C	THF	*3.02*	Lehmann et al. (1995)
HP-5 MS,<br>*30 x 0.25 x 0.25*	He,<br>n.a.	70 °C 3 min,<br>20 °C/min up to 210 °C	THF<br><br>acetone	*3.04*<br><br>*2.43*	Varaprath and Lehmann (1997)
n.a.	n.a	n.a.	n.a.	3.02	Varaprath (1999)
n.a.	n.a.	n.a.	n.a.	~1.84	Cabrera-Codony et al. (2015)
Rxi-5ms,<br>*30 x 0.25 x 0.25*	He,<br>1	45 °C 5 min,<br>5 °C/min up to 250 °C	--	*2.47*<br>*2.49*<br>*2.52*	Niu et al. (2016)
HP-5ms Ultra Inert,<br>dimensions n.a.	n.a.<br>n.a.	60 °C 3 min,<br>20 °C/min up to 120 °C,<br>40 °C/min up to 250 °C	THF	2.2	Cabrera-Codony et al. (2017)
HP-5 MS,<br>*30 x 0.25 x 0.5*	He,<br>(9.4 psi)	60 °C,<br>3 °C/min up to 240 °C	--	*3.51*	Babatunde et al. (2019)
DB-624,<br>30 x 0.32 x 1.8	n.a.<br>2.9	50 °C 2 min,<br>40 °C/min up to 240 °C	THF	4.1	Xu (2019)

Zebron 5MSi,<br>*30 x 0.25 x 0.25*	He,<br>1.5	30 °C 5 min,<br>20 °C/min up to 300 °C	n.a.	*4.301*	Konarzewska et al. (2020)
PDMS,<br>30 x 0.25 x ?? or 50 x 0.25 x ??	N_2,<br>1	80 °C 1 min,<br>5 °C/min up to 200 °C	H_2O<br>MeOH/H_2O	3.109<br>3.239	Msiska et al. (2020)
DB-624,<br>30 x 0.32 x 1.8	He,<br>1	n.a.	THF	6.22	Liu et al. (2021)
Restek Rxi-624,<br>30 x 0.25 x 1.4	He,<br>2	40 °C 40 s,<br>40 °C/min up to 100 °C,<br>15 °C/min up to 150 °C,<br>30 °C/min up to 225°C	--	3.83	Claflin et al. (2021)
HP-5ms,<br>*30 x 0.25 x 0.25*	He,<br>1	50 °C 1 min,<br>10 °C/min up to 280 °C	--	*3.036*	Tu et al. (2022)

References to Table S1

Lehmann RG, Varaprath S, Frye CL (1994) Fate of silicone degradation products (silanols) in soil. Environ Toxicol Chem 13:1753-1759. https://doi.org/10.1002/etc.5620131106

Lehmann RG, Varaprath S, Annelin RB, Arndt JL (1995) Degradation of silicone polymer in a variety of soils. Environ Toxicol Chem 14:1299-1305. https://doi.org/10.1002/etc.5620140806

Varaprath S, Lehmann RG (1997) Speciation and quantitation of degradation products of silicones (silane/siloxane diols) by gas chromatography-mass spectrometry and stability of dimethylsilanediol. J Environ Polym Degrad 5:17-31. https://doi.org/10.1007/BF02763565

Varaprath S (1999) Synthesis of ^{14}C-labeled cyclic and linear siloxanes. J Organomet Chem 572:37-47. https://doi.org/10.1016/S0022-328X(98)00916-4

Cabrera-Codony A, Gonzalez-Olmos R, Martin MJ (2015) Regeneration of siloxane-exhausted activated carbon by advanced oxidation processes. J Hazard Mater 285:501-508. https://doi.org/10.1016/j.jhazmat.2014.11.053

Niu Y, Hua L, Hardy G, Agarwal M, Ren Y (2016) Analysis of volatiles from stored wheat and *Rhyzopertha dominica* (F.) with solid phase microextraction–gas chromatography mass spectrometry. J Sci Food Agriculture 96:1697–1703. https://doi.org/10.1002/jsfa.7274

Cabrera-Codony A, Georgi A, Gonzalez-Olmos R, Valdes H, Martin MJ (2017) Zeolites as recyclable adsorbents/catalysts for biogas upgrading: Removal of octamethylcyclotetrasiloxane. Chem. Eng. J. 307:820-827. https://doi.org/10.1016/j.cej.2016.09.017

Babatunde DE, Otusemade GO, Efeovbokhan VE, Ojewumi ME, Bolade OP, Owoeye TF (2019) Chemical composition of steam and solvent crude oil extracts from *Azadirachta indica* leaves. Chem Data Collections 20:100208. https://doi.org/10.1016/j.cdc.2019.100208

Xu S (2019) Extraction and quantitative analysis of water by GC/MS for trace-level dimethylsilanediol (DMSD). J Chromatogr A 1600:1-8. https://doi.org/10.1016/j.chroma.2019.04.026

Konarzewska Z, Sliwinska-Wilczewska S, Felpeto AB, Vasconcelos V, Latala A (2020) Assessment of the allelochemical activity and biochemical profile of different phenotypes of picocyanobacteria from the genus *Synechococcus*. Mar Drugs 2020, 18:179. https://doi.org/10.3390/md18040179

Msiska T, Mwakikunga A, Tembo D, Lampiao F (2020) A phytochemical analysis and *in vivo* effects of an herbal aphrodisiac *Newtonia hildebrandtii* on male Wistar rat reproductive system. Pharmacognosy Res. 12:243-249. 10.4103/pr.pr_112_19

Liu N, Sun H, Xu L, Cai Y (2021) Methylsiloxanes in petroleum refinery facility: Their sources, emissions, environmental distributions and occupational exposure. Environ Int 152:106471. https://doi.org/10.1016/j.envint.2021.106471

Claflin MS, Pagonis D, Finewax Z, Handschy AV, Day DA, Brown WL, Jayne JT, Worsnop DR, Jimenez JL, Ziemann PJ, de Gouw J, Lerner BM (2021). An in situ gas chromatograph with automatic detector switching between PTR- and EI-TOF-MS: isomer-resolved measurements of indoor air. Atmos Meas Tech 14:133–152. https://doi.org/10.5194/amt-14-133-2021

Tu X, Liu Y, Yanli Y, Wenxiu L, Ping L, Du L, He J, Jian-neng L (2022). Effects of four drying methods on *Amomum villosum* Lour. 'Guiyan1' volatile organic compounds analyzed *via* headspace solid phase microextraction and gas chromatography-mass spectrometry coupled with OPLS-DA. RSC Advances 12:26485-26496. https://doi.org/10.1039/D2RA04592C

Table S2. GC columns, conditions and retention times of L_2

**GC Column material, dimensions** (m x mm x μm)	**Carrier gas, flow rate** (mL/min)	**Temperature program**	**Solvent**	**L_2 retention time (min)**	**Reference**
n.a.	n.a.	n.a.	n.a.	3.88	Varaprath (1999)
HP5, 30 x 0.25 x 1.0	He, 1.1	40 °C 2 min, 5 °C/min up to 150 °C, 15 °C/min up to 250 °C	--	*6.4*	Rasi et al. (2010)
HP-5MS, 30 x 0.25 x 0.25	He, ~1	70 °C 1 min, 25 °C/min up to 160 °C	--	*1.8687*	Ajhar et al. (2010)
DB-5MS, 60 x 0.25 x 0.25	He, 1	40 °C 2 min, 10 °C/min up to 250 °C	--	5.4	Companioni-Damas et al. (2012)
HP5, 30 x 0.25 x 1.0	He, 1.1	40 °C 2 min, 5 °C/min up to 150 °C, 15 °C/min up to 250 °C	--	*6.4*	Läntelä et al. (2012)

HP1-MS,<br>30 x 0.25 x 0.25	He,<br>1	n.a.	MeOH	2.3	Kim et al. (2013)
HP5,<br>30 x 0.25 x 1.0	He,<br>1.1	40 °C 2 min,<br>5 °C/min up to 150 °C,<br>15 °C/min up to 250 °C	--	*6.4*	Rasi et al. (2013)
DB-624,<br>30 x 0.25 x 1.4	He,<br>n.a.	40 °C 2 min,<br>12 °C/min up to 60 °C,<br>25 °C/min up to 240 °C	n-hexane	5.6	Raich-Montiu, et al. (2014)
HP-5MS,<br>30 x 0.25 x 0.25	He,<br>n.a.	40 °C 2 min,<br>12 °C/min up to 60 °C,<br>25 °C/min up to 240 °C	n-hexane	*~2.2*	Raich-Montiu, et al. (2014)
DB-624,<br>60 x 0.25 x 1.4	He,<br>1	40 °C 2 min,<br>6 °C/min up to 120 °C,<br>120 °C 5 min,<br>8 °C/min up to 150 °C,<br>20 °C/min up to 220 °C	C_6H_5Cl	12.6	Cortada et al. (2014)
DB-5MS,<br>60 x 0.25 x 0.25	He,<br>1	60 °C 5 min,<br>15 °C/min up to 285 °C	n-hexane	8.4-9.2	Companioni-Damas et al. (2014)
DB-624,<br>60 x 0.25 x 1.4	He,<br>1	40 °C 1 min,<br>6 °C/min up to 230 °C	--	~9.2	Gallego et al. (2015)
Carbowax,<br>30 x 0.32 x 0.25	He,<br>2	60 °C,<br>10 °C/min up to 210 °C	acetone	1.933	Piechota et al. (2015)
Rxi-5MS,<br>60 x 0.25 x 1.0	He,<br>2.43	50 °C 5 min,<br>5 °C/min up to 200 °C	--	10.17	Salazar Gomez et al. (2016)
DB-5 ms ultra inert,<br>30 x 0.25 x 0.25	He,<br>1	35 °C 5 min,<br>10 °C/min up to 160 °C	n-hexane	*3.48*	Ramos et al. (2016)
CP-SIL 8-CB,<br>50 x 0.25 x 0.12	He,<br>1	35 °C 5 min,<br>6 °C/min up to 155 °C,<br>20 °C/min up to 300 °C	n-hexane	5.75	Capela et al. (2016)
Elite-5ms,<br>60 x 0.25 x 1	He,<br>n.a.	30 °C 1 min,<br>20 °C/min up to 300 °C	--	5.85	Delzeit and Hunter (2018)
HP5,<br>30 x 0.25 x 0.25	He,<br>1	36 °C 5 min,<br>7.5 °C/min up to 200 °C,	--	*2.93*	Ghidotti et al. (2019)

		100 °C/min up to 300 °C			
TR-1MS,<br>30 x 0.25 x 0.25	n.a.<br>n.a.	50 °C 1 min,<br>15 °C/min up to 180 °C	--	~2.1	Feng et al. (2019)
HP-5 MS,<br>30 x 0.25 x 0.25	He,<br>1.5	50 °C,<br>25 °C/min up to 180 °C,<br>10 °C/min up to 300 °C	n-hexane	*~1.65*	Feng et al. (2019)
InoWax,<br>30 x 0.25 x 0.25	n.a.	60 °C 30 min,<br>10 °C/min up to 250 °C	n.a.	2.513	Chen et al. (2019)
DB-5MS,<br>dimensions n.a.	He,<br>n.a.	40 °C 2 min,<br>20 °C/min up to 100 °C,<br>100 °C 3 min,<br>10 °C/min up to 200 °C	MeOH	3.22	Wang et al. (2020)
HP5,<br>30 x 0.32 x 0.25	He,<br>2	55 °C 3 min,<br>20 °C/min up to 200 °C	2-propanol	*~1.9*	Foppiano et al. (2020)
DB-5 MS UI,<br>30 x 0.25 x 0.25	He,<br>0.5	35 °C 5 min,<br>10 °C/min up to 140 °C,<br>20 °C/min up to 250 °C	FCC gasoline	*3.27*	Sanchez et al. (2020)
PDMS,<br>30 x 0.25 x 0.25	He,<br>2	50 °C 10 min,<br>up to 220 °C	acetone/<br>MeOH (1/1)	1.631	Piechota (2021)
DB-624 UI,<br>30 x 0.25 x 1.4	He,<br>1.5	40 °C 2 min,<br>12 °C/min up to 60 °C,<br>25 °C/min up to 240 °C	acetone	5.5	Wang et al. (2022)

References to Table S2

Varaprath S (1999). Synthesis of ^{14}C-labeled cyclic and linear siloxanes. J Organomet Chem 572:37-47. https://doi.org/10.1016/S0022-328X(98)00916-4

Rasi S, Lehtinen J, Rintala J (2010). Determination of organic silicon compounds in biogas from wastewater treatments plants, landfills, and co-digestion plants. Renew Energy 35:2666-2673. https://doi.org/10.1016/j.renene.2010.04.012

Ajhar M, Wens B, Stollenwerk KH, Spalding G, Yüce S, Melin T (2010) Suitability of Tedlar gas sampling bags for siloxane quantification in landfill gas. Talanta 82:92-98. https://doi.org/10.1016/j.talanta.2010.04.001

Companioni-Damas EY, Santos FJ, Galceran MT (2012) Analysis of linear and cyclic methylsiloxanes in water by headspace-solid phase microextraction and gas chromatography-mass spectrometry. Talanta 89:63-69. https://doi.org/10.1016/j.talanta.2011.11.058

Läntelä J, Rasi S, Lehtinen J, Rintala J (2012) Landfill gas upgrading with pilot-scale water scrubber: Performance assessment with absorption water recycling. Applied Energy 92:307-314. https://doi.org/10.1016/j.apenergy.2011.10.011

Kim N, Chun S, Cha DK, Kim C (2013) Determination of siloxanes in biogas by solid-phase adsorption on activated carbon. Bull Korean Chem Soc 34:2353-2357. https://doi.org/10.5012/bkcs.2013.34.8.2353

Rasi S, Seppälä M, Rintala J (2013) Organic silicon compounds in biogases produced from grass silage, grass and maize in laboratory batch assays. Energy 52:137-142. https://doi.org/10.1016/j.energy.2013.01.015

Raich-Montiu J, Ribas-Font C, de Arespacochaga N, Roig-Torres E, Broto-Puig F, Crest M, Bouchy L, Cortina JL (2014) Analytical methodology for sampling and analysing eight siloxanes and trimethylsilanol in biogas from different wastewater treatment plants in Europe. Anal Chim Acta 812:83-91. https://doi.org/10.1016/j.aca.2013.12.027

Cortada C, Costa dos Reis L, Vidal L, Llorca J, Canals A (2014). Determination of cyclic and linear siloxanes in wastewater samples by ultrasound-assisted dispersive liquid-liquid microextraction followed by gas chromatography-mass spectrometry. Talanta 120:191-197. https://doi.org/10.1016/j.talanta.2013.11.042

Companioni-Damas EY, Santos FJ, Galceran MT (2014) Linear and cyclic methylsiloxanes in air by concurrent solvent recondensation-large volume injection-gas chromatography-mass spectrometry. Talanta 118:245-252. https://doi.org/10.1016/j.talanta.2013.10.020

Gallego E, Roca FJ, Perales JF, Guardino X, Gadea E (2015) Development of a method for determination of VOCs (including methylsiloxanes) in biogas by TD-GC/MS analysis using Supel™ Inert Film bags and multisorbent bed tubes. Int J Environ Anal Chem 95:291-311. https://doi.org/10.1080/03067319.2015.1016012

Piechota G, Iglinski B, Buczkowski R (2015) An experimental approach for the development of direct-absorption sampling method for determination of trimethylsilanol and volatile methylsiloxanes by the GC-MS technique in landfill gas. Int J Environ Anal Chem 95:867–877. https://doi.org/10.1080/03067319.2015.1055473

Salazar Gomez JI, Lohmann H, Krassowski J (2016) Determination of volatile organic compounds from biowaste and cofermentation biogas plants by single-sorbent adsorption. Chemosphere 153:48-57. https://doi.org/10.1016/j.chemosphere.2016.02.128

Ramos S, Silva JA, Homem V, Cincinelli A, Santos L, Alves A, Ratola N (2016) Solvent-saving approaches for the extraction of siloxanes from pine needles, soils and passive air samplers. Anal Methods 8:5378-5387. https://doi.org/10.1039/C6AY00506C

Capela D, Alves A, Homem V, Santos L (2016) From the shop to the drain — Volatile methylsiloxanes in cosmetics and personal care products. Environ Int 92–93:50–62. https://doi.org/10.1016/j.envint.2016.03.016

Delzeit L, Hunter CJ (2018) UV chemistry and mitigation of siloxane. 48th International Conference on Environmental Systems ICES-2018-284, 8-12 July 2018, Albuquerque, NM.

Ghidotti M, Fabbri D, Torri C (2019) Determination of linear and cyclic volatile methyl siloxanes in biogas and biomethane by solid-phase microextraction and gas chromatography-mass spectrometry. Talanta 195:258-264. https://doi.org/10.1016/j.talanta.2018.11.032

Feng D, Zhang X, Wang W, Li Z, Cao X (2019) Development, validation and comparison of three detection methods for 9 volatile methylsiloxanes in food-contact silicone rubber products. Polymer Testing 73:94-103. https://doi.org/10.1016/j.polymertesting.2018.10.014

Chen W, Luo Z, Wu C, Wen P, Li Q (2019) Oxidative removal of recalcitrant organics in shale gas flowback fluid by the microwave-activated persulfate process. Environ Sci Pollut Res 26:684-693. https://doi.org/10.1007/s11356-018-3668-5

Wang N, Tan L, Xie L, Wang Y, Ellis T (2020) Investigation of volatile methyl siloxanes in biogas and the ambient environment in a landfill. J Environ Sci 91:54-61. https://doi.org/10.1016/j.jes.2020.01.005

Foppiano D, Tarik M, Schneebeli J, Calbry-Muzyka A, Biollaz S, Ludwig C (2020). Siloxane compounds in biogas from manure and mixed organic waste: Method development and speciation analysis with GC-ICP-MS. Talanta 208:120398. https://doi.org/10.1016/j.talanta.2019.120398

Sanchez R, Chainet F, Souchon V, Carbonneaux S, Lienemann CP, Todoli JL (2020). Silicon speciation in light petroleum products using gas chromatography coupled to ICP-MS/MS. J Anal At Spectrom 35:2387-2394. https://doi.org/10.1039/D0JA00156B

Piechota G (2021) Removal of siloxanes from biogas upgraded to biomethane by cryogenic temperature condensation system. J Cleaner Prod 308:127404. https://doi.org/10.1016/j.jclepro.2021.127404

Wang J, Liao L, Wang L, Wang L (2022) Influence of sampling methods and storage condition on volatile methyl siloxanes quantification in biogas. Biomass Bioenergy 158:106347. https://doi.org/10.1016/j.biombioe.2022.106347
